# Predictive Value of Mean Platelet Volume in Saphenous Vein Graft Disease

**DOI:** 10.21470/1678-9741-2017-0247

**Published:** 2018

**Authors:** Ugur Kaya, Yavuzer Koza

**Affiliations:** 1 Department of Cardiovascular Surgery, Ataturk University, Faculty of Medicine, Erzurum, Turkey.; 2 Department of Cardiology, Ataturk University, Faculty of Medicine, Erzurum, Turkey.

**Keywords:** Saphenous Vein/Pathology/*Transplantation, Hyperplasia, Thrombosis, Arteriosclerosis, Mean Platelet Volume, Coronary Artery Bypass

## Abstract

**Objective:**

To determine whether mean platelet volume (MPV), platelet distribution width (PDW), and platelet count could be used as determinants of mortality following coronary artery bypass graft (CABG) surgery and patency of saphenous vein grafts (SVG).

**Methods:**

The records of 128 patients who underwent emergency or elective coronary angiography after CABG surgery, and who died at an early stage were retrospectively reviewed. Patients were divided into three groups as early death, no SVG disease (SVGD), and SVGD group. MPV, PDW, and platelet count were evaluated at different times.

**Results:**

MPV was significantly higher in the stenotic group than in the nonstenotic group (9.7±1.8 fl and 8.2±0.9 fl, *P*<0.05). The postoperative MPV ratio was found to be higher in the stenotic group when compared to the preoperative period (9.6±1.8 fl and 7.8±0.9 fl, *P*<0.05). MPV values were also found to be higher in patients who died during the early stage than in surviving patients (9.4±1.9 fl and 8.0±1.0 fl, *P*<0.05). There was no statistically significant difference regarding platelet count and PDW ratios between the early deaths group and surviving patients. An MPV value higher than 10.6 predicted SVGD with 85% sensitivity and 45% specificity; and an MPV higher than 7.9 predicted early death with 80% sensitivity and 68% specificity were observed.

**Conclusion:**

MPV may be a useful indicator for the prediction of SVGD and mortality following CABG surgery.

**Table:** 

Abbreviations, acronyms & symbols	
ASA	= Acetylsalicylic acid
CABG	= Coronary artery bypass graft
EDTA	= Ethylenediaminetetraacetic acid
EF	= Ejection fraction
LIMA	= Left internal mammary artery
MPV	= Mean platelet volume
PDW	= Platelet distribution width
ROC	= Receiver operating characteristic
SD	= Standard deviation
SVG	= Saphenous vein grafts
SVGD	= Saphenous vein grafts disease

## INTRODUCTION

Coronary artery bypass graft (CABG) surgery is a commonly used treatment method for stenotic coronary artery revascularization. Saphenous veins and arterial grafts are widely used in CABG surgery. The patency rates of saphenous vein grafts (SVG) are relatively low. A 10-year patency rate for SVG was reported as 61%^[1]^.

Different processes have been proposed for SVG disease (SVGD), including thrombosis, intimal hyperplasia, and atherosclerosis. The contribution of these processes may differ among patients. Therefore, many studies have been conducted to identify risk indicators during SVGD. Platelets are known to play an important role in the pathophysiology of coronary artery disease and SVGD. The main effect of platelets known to have activity in atherogenesis, which begins with the endothelial injury, is atherothrombosis, a dangerous complication in the advanced stage of atherosclerosis^[2]^. Mean platelet volume (MPV) is an important indicator of platelet activation. Platelet distribution width (PDW) and platelet count have also been associated with SVGD^[2-4]^.

We aimed to determine whether MPV and PDW values and platelet count could be used as predictive parameters for early death after CABG surgery and graft occlusion during SVGD.

## METHODS

This retrospective study included 128 patients (77 males, 51 females) who underwent emergency or elective coronary angiography after CABG surgery, between 2010 and 2017, and who died during the early stages (deﬁned as in-hospital or 60-day mortality). All operations were performed by a single surgical team. Patients were divided into three groups according to SVG patency and early death: Group I, early death (n=30, 19 males, 11 females, mean age 62.3±9.6); Group II, nonstenotic group (n=49, 28 males, 21 females, mean age 59.9±10.6); and Group III, stenotic group (SVGD) (n=49, 30 males, 19 females, mean age 61.7±11.7). Coronary angiographies of patients were assessed by two independent examiners. Stenosis of 50% or more in SVG was considered hemodynamically significant. Patients underwent CABG operation included at least the use of one SVG. The left internal mammary artery (LIMA) was used in all patients, except eight.

Indications for coronary angiography were as follows: recurrent postoperative stable angina pectoris, unstable angina pectoris, myocardial infarction, and preoperative evaluation for non-cardiac surgeries. Before the angiography procedure, medical history and risk factors for coronary heart disease of all patients were evaluated. Patients with SVG and problems in the native artery anastomosis path, those with severe cardiac valve disease and other diseases with cardiac manifestations (*e.g.,* ventricular septal rupture and dissection), patients with renal and hepatic dysfunction, patients with acute and chronic infections, and patients with bone marrow problems were excluded from the study. Pre and postoperative MPV and PDW values and platelet counts were obtained from hospital records and compared among all groups. Patients included in the study were also evaluated with regards to risk factors, such as age, gender, cigarette smoking, diabetes mellitus, hypertension, hyperlipidemia, drug use, ejection fraction (EF), creatinine clearance, and survival after surgery.

An approval of the local Ethics Committee was obtained for this study. A written informed consent was obtained from each patient. The study was conducted in accordance with the principles of the Declaration of Helsinki. Demographic information, postoperative data, and operative details were retrospectively collected from medical records and the Hospital Electronic Record System.

Laboratory Analysis: MPV-d, PDW-d, and platelet count-d values were measured at the time of diagnosis (d). MPV-ca, PDW-ca, and platelet count-ca values were assessed at the time of control angiography (ca).

The blood samples were obtained using ethylenediaminetetraacetic acid (EDTA) for anticoagulation. MPV and PDW values were analyzed with aperture-impedance technology, using the Beckman Coulter Gen-S (Beckman Coulter Corporation, Miami, USA; GEN-S) device. Impedance reference values were determined as 6.8-10.8 fl for MPV and 10.0-14.0 fl for PDW.

### Statistical Analysis

Statistical analysis was performed using SPSS package (version 20.0; SPSS for Windows, Chicago, IL, USA). Descriptive statistics were reported, including mean, standard deviation (SD), median, interquartile range, and percentage. The t-test was used in comparisons of the deceased and surviving patients; it was also used in comparisons of patient groups with and without SVGD. Receiver operating characteristic (ROC) curves were analyzed to assess the optimal cut-off values of influence factors. Sensitivity and specificity were calculated for the chosen cut-off values. *P*<0.05 was considered statistically significant.

## RESULTS

A total of 128 patients were evaluated in this study; 77 (60.2%) males and 51 (39.8%) females. Demographic characteristics of these patients and laboratory results are shown in Table 1. Low EF value and cigarette smoking in the stenotic (SVGD) group were found to be statistically significant (0.043 and 0.016, respectively; *P*<0.05).

**Table 1:** Baseline clinical characteristics of patients with or without saphenous vein graft disease.

	Nonstenotic group (n=49)	Stenotic group (n=49)	*P* value
Age (mean ± SD)	59.9±10.6	61.7±11.7	0.39
Sex (male/female)	Males 28 (57.1%)	Males 30 (61.2%)	0.18
	Females 21 (42.9%)	Females 19 (38.8%)	0.15
Time of angiography after surgery	2.9±2.5 (years)	3.5±2.5 (years)	0.16
Hypertension	20 (40.8%)	22 (44.8%)	0.13
Diabetes mellitus	28 (57.1%)	31 (63.3%)	0.79
Smoking	8 (16.1%)	17 (34.7%)	0.016
Hypercholesterolemia	18 (36.7%)	21 (42.9%)	0.81
Creatinine (mg/dL)	1.09±0.9	1.17±0.4	0.92
Ejection fraction (%)	58.3±5.3	50.21±9.2	0.043
Aspirin	43 (87.8%)	45 (91.8%)	0.95
Statins	26 (53.1%)	30 (61.2%)	0.89
Internal mammary artery graft	45 (91.8%)	46 (93.9%)	0.18
Saphenous vein grafts per patients	2.3±0.9	2.6±0.9	0.21
EuroSCORE	4.16±2.79	4.30±2.89	0.36

SD=standard deviation

**Fig. 1:**
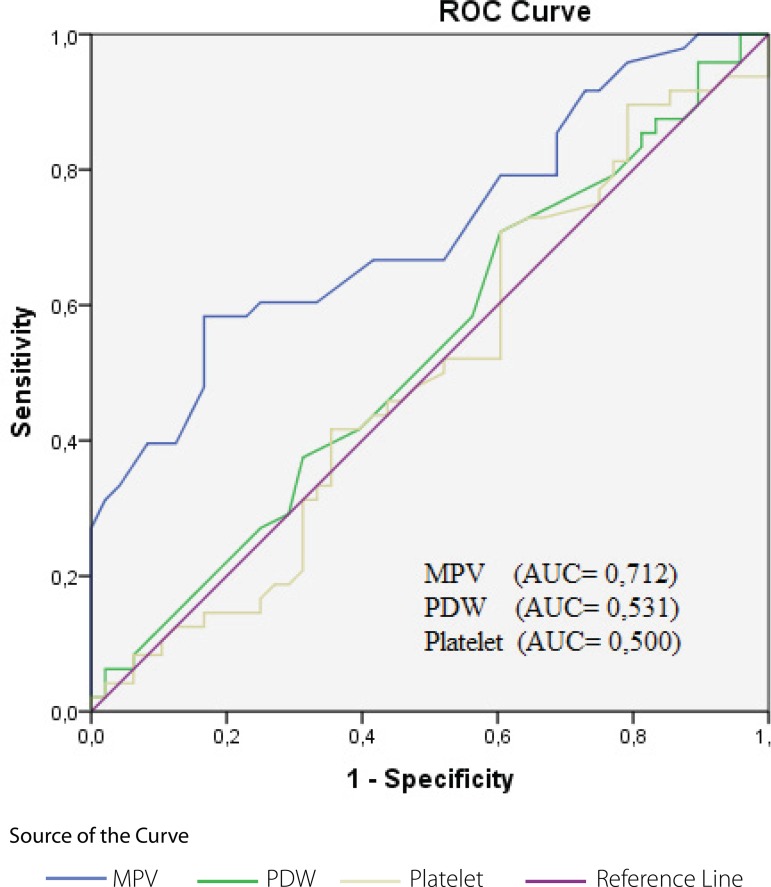
The receiver operating characteristic (ROC) curve of postoperative MPV, PDW, and platelet count ratio for the prediction of saphenous vein graft disease (stenosis group). Occlusion was determined by coronary angiography artery within 3.2±2.4 years after CABG. AUC=area under the curve; CABG=coronary artery bypass graft; MPV=mean platelet volume; PDW=platelet distribution width

**Fig. 2:**
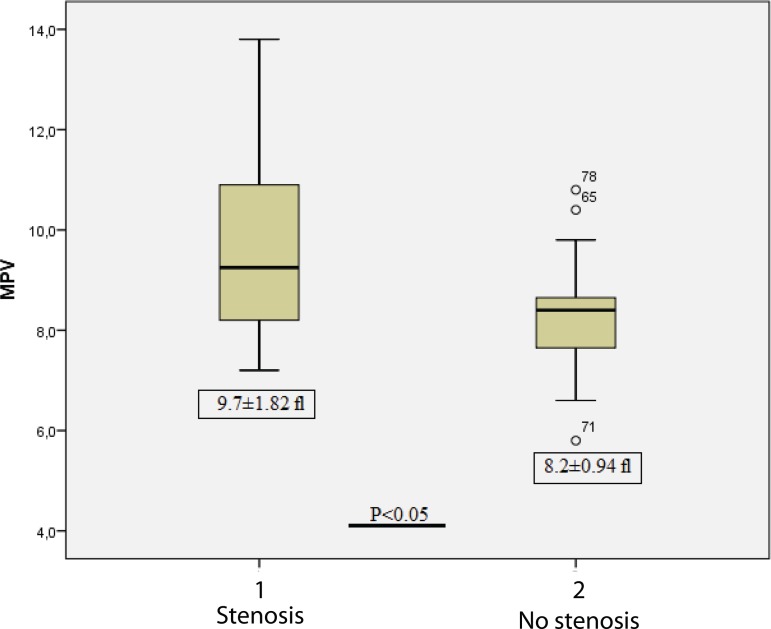
Comparison of mean platelet volume (MPV) in stenotic and nonstenotic groups.

Comparisons were made between patients with stenosis (SVGD) and no stenosis regarding MPV-d and MPV-ca; PDW-d and PDW-ca; and platelet count-d and platelet count-ca values. MPV-ca value was found to be statistically significantly higher in the stenotic group (Group III) than in the nonstenotic group (Group II) (9.7±1.82 and 8.2±0.94, respectively; *P*<0.05). PDW-ca value was 16.3±0.9 in the nonstenotic group (Group II) and 16.5±1.5 in the stenotic group (Group III); results were found to be statistically insignificant (*P*=0.43) (Table 2). Comparison of the preoperative (at the time of diagnosis) and postoperative (at the time of control angiography) values in the stenotic group (Group III) demonstrated that the high MPV value during control angiography was statistically significant about the preoperative period (9.6±1.8 and 7.8±0.9, respectively; *P*<0.05) (Table 3). ROC curve for MPV, PDW, and platelet count in patients with stenosis (SVGD, Group III) is shown in Figure 1 and the comparison of MPV values in the nonstenotic patients (Group II) is shown in Figure 2. An MPV value higher than 10.6 fl had 85% sensitivity and 45% specificity.

**Table 2:** Comparison of patients with or without saphenous vein graft disease.

	Nonstenotic group (n=49)	Stenotic group (n=49)	*P* value
MPV-d	8.1±1.13	7.9±0.99	0.37
MPV-ca	8.2±0.94	9.7±1.82	0.0003
PDW-d	16.7±0.94	16.7±1.11	0.75
PDW-ca	16.3±1.83	16.5±1.52	0.42
Platelet count-d	249,000±101,500	272,000±93,000	0.26
Platelet count-ca	224,000±67,000	221,000±68,200	0.81

MPV-ca=mean platelet volume (control angiography); MPV-d=mean platelet volume (diagnosis); PDW-ca=platelet distribution width (control angiography); PDW-d=platelet distribution width (diagnosis)

**Table 3:** Comparison of preoperative and postoperative MPV, PDW, and platelet count of patients with saphenous vein graft disease (stenosis group).

	Preoperative	Postoperative	*P* value
MPV	7.8±0.9	9.6±1.8	0.0001
PDW	16.6±0.9	16.5±1.5	0.59
Platelet count	221,000±68,200	272,000±93,300	0.003

MPV=mean platelet volume; PDW=platelet distribution width

**Fig. 3:**
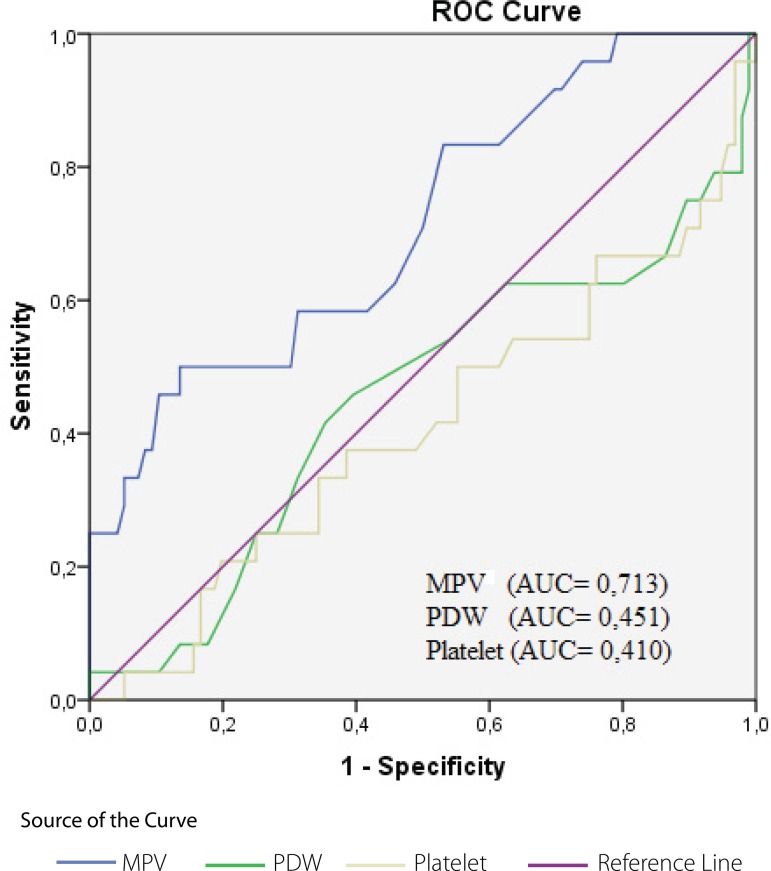
MPV and PDW values and platelet count of deceased patients at the time of diagnosis. AUC=area under the curve; MPV=mean platelet volume; PDW=platelet distribution width; ROC=receiver operating characteristic

A comparison was made between patients who died at an early stage and surviving patients regarding MPV-d, PDW-d, and platelet count-d values. MPV-d values in the early death group were found to be higher than in surviving patients (9.4±1.9 fl and 8.0±1.0 fl, respectively; *P*<0.05). There was no statistically significant difference with regards to PDW-d and platelet count-d values (*P*>0.05) (Table 4). ROC curves for MPV-d, PDW-d, and platelet count-d in the early death group are shown in Figure 3. An MPV value higher than 7.9 fl had 80% sensitivity and 68% specificity.

**Table 4:** Comparison of MPV, PDW, and platelet count of deceased and surviving patients.

	Early death (n=30)	Surviving patients (n=98)	*P* value
MPV-d	9.4±1.9	8.0±1.0	0.0001
PDW-d	16.9±1.1	15.8±2.1	0.08
Platelet count-d	248,000±97,600	260,000±97,600	0.2

MPV-d=mean platelet volume (diagnosis); PDW-d=platelet distribution width (diagnosis)

## DISCUSSION

CABG surgery has been accepted as one of the most effective treatment modalities for coronary artery disease. However, long-term outcomes are limited to SVGD^[5]^. Several risk factors, such as hypertension, diabetes mellitus, cigarette smoking, hyperlipidemia, native vessel diameter, age of the graft, severity of bypassed proximal vessel stenosis, plasma lipoprotein (a), homocysteine, and fibrinogen levels, have been identified for SVGD. In addition, harvesting of venous grafts has been found to be associated with endothelial injury during or after surgery, which may also contribute to SVGD^[6,7]^. Unlike with arterial grafts, venous grafts are more sensitive to intimal hyperplasia, atherosclerosis, progressive stenosis, and occlusion. All stages of the pathological process are closely related to platelet and platelet functions. Although the role of platelets in the vein graft disease has been evaluated, their relationship has not yet been classified^[8]^.

Steele et al.^[8]^ demonstrated the relationship between occlusion of the saphenous vein graft and platelet viability. Similarly, Latour et al.^[9]^ found platelet regeneration time reduced in patients with saphenous bypass graft. Enzymatic and metabolic activity of the platelets was reported to increase with an augmentation in the platelet volume. Vasoactive mediators secreted by the platelets may contribute to inflammation and the process of atherogenesis. Increased risk of acute coronary syndrome in patients with high MPV may be explained by increased platelet activity, inflammation, and platelet aggregation^[10]^. MPV has been found to be more closely related to platelet function than to platelet count. In addition to the platelet count, several factors were shown to affect platelet function, including platelet size, density, age, and previous hemostatic interactions^[11]^.

The increase in MPV may be due to the depletion of small platelets during ischemia^[11]^. Hemostatically reactive platelets, with more granular and larger platelets with adhesion receptors resulting in decreased bleeding time, showed an increased activity^[12]^. As a result, in addition to reflecting platelet count, MPV also demonstrated platelet activity, and it was considered a determining factor in atherosclerotic coronary disease^[13]^. Many studies have also shown that increased platelet activation and aggregation are closely correlated with cardiovascular complications^[3,6,8,9]^. Tavil et al.^[3]^ found that MPV values were higher in the stenotic group when they compared patients with SVGD and without SVGD, according to the results of coronary angiography after coronary bypass surgery, and they identified MPV as a possible postoperative indicator of graft success.

In the present study, we found MPV values significantly high during the development of SVGD and in early (premature) death. This finding may be attributed to intimal hyperplasia in patients with SVGD and to the result of atherosclerosis in increased thrombosis, causing megakaryocyte and platelet activities increased in the bone marrow of the ongoing thrombotic event. High platelet volume in patients with SVGD and in those with premature death supports this notion. We could not demonstrate any difference with regards to PDW and platelet count.

Platelet activation and venous graft thrombosis are considered early steps in the stenosis and occlusion process after CABG surgery. Although many studies have shown the efficacy of antiplatelet therapy during postoperative period, there is still no consensus on the dose of this treatment and whether it should be used as a single acetylsalicylic acid (ASA) drug or as combination therapy (ASA + Clopidogrel)^[14]^. In our study, high MPV values in patients with mortality and stenosis suggest that antiplatelet therapy should be widely used in these patients during postoperative period.

In the present study, the difference in EF between the groups can be associated with SVG occlusion as previously shown^[15]^. This suggests that poor left ventricular function contributes to SVGD.

### Limitation

The main limitation of this study was its small sample size with a retrospective design. However, the inclusion of patients with premature death after CABG surgery and those with acute coronary syndrome and the presence of diagnostic and postoperative data were the advantages of this study.

## CONCLUSION

The present study revealed a cut-off MPV value for the prediction of SVG patency after CABG surgery. We believe that MPV could be used as a determinant of graft patency and mortality after this surgery. Furthermore, MPV values can be considered when deciding on the intensity of postoperative antiplatelet therapy.

**Table:** 

Authors' roles & responsibilities	
UK	Substantial contributions to the conception or design of the study; or the acquisition, analysis, or interpretation of data for the study; final approval of the version to be published
YK	Final approval of the version to be published
